# Novel subscalp and intracranial devices to wirelessly record and analyze continuous EEG in unsedated, behaving dogs in their natural environments: A new paradigm in canine epilepsy research

**DOI:** 10.3389/fvets.2022.1014269

**Published:** 2022-10-20

**Authors:** Wolfgang Löscher, Gregory A. Worrell

**Affiliations:** ^1^Department of Pharmacology, Toxicology, and Pharmacy, University of Veterinary Medicine, Hanover, Germany; ^2^Center for Systems Neuroscience, Hanover, Germany; ^3^Bioelectronics Neurophysiology and Engineering Laboratory, Department of Neurology, Mayo Clinic, Rochester, MN, United States; ^4^Department of Physiology and Biomedical Engineering, Mayo Clinic, Rochester, MN, United States

**Keywords:** seizures, dogs, anti-seizure medication, seizure diaries, biomarkers, ambulatory EEG

## Abstract

Epilepsy is characterized by unprovoked, recurrent seizures and is a common neurologic disorder in dogs and humans. Roughly 1/3 of canines and humans with epilepsy prove to be drug-resistant and continue to have sporadic seizures despite taking daily anti-seizure medications. The optimization of pharmacologic therapy is often limited by inaccurate seizure diaries and medication side effects. Electroencephalography (EEG) has long been a cornerstone of diagnosis and classification in human epilepsy, but because of several technical challenges has played a smaller clinical role in canine epilepsy. The interictal (between seizures) and ictal (seizure) EEG recorded from the epileptic mammalian brain shows characteristic electrophysiologic biomarkers that are very useful for clinical management. A fundamental engineering gap for both humans and canines with epilepsy has been the challenge of obtaining continuous long-term EEG in the patients' natural environment. We are now on the cusp of a revolution where continuous long-term EEG from behaving canines and humans will be available to guide clinicians in the diagnosis and optimal treatment of their patients. Here we review some of the devices that have recently emerged for obtaining long-term EEG in ambulatory subjects living in their natural environments.

## Introduction

Epilepsy, which is characterized by unprovoked (spontaneous) recurrent seizures, is the most frequent brain disease in domestic dogs (1–3). Although the actual prevalence of canine epilepsy in the overall dog population is unclear, it has been estimated to be between 0.6 and 0.7% (4, 5). Greater prevalence rates than those observed in the general dog population have been documented in dog breeds prone to idiopathic epilepsy, which is one of the reasons a hereditary component is thought to be present in some dog breeds (6). As in humans (7), canine epilepsy is not a single illness but rather a collection of illnesses with a wide range of clinical symptoms, onset ages, and underlying causes (8). In a recent epidemiologic study in 900 dogs undergoing magnetic resonance imaging (MRI) for recurrent seizures, structural lesions were identified as a cause of seizures in 45.1% (*n* = 406) of cases (9). Dogs with epilepsy (DWE) and structural lesions are categorized as having structural (or symptomatic) epilepsy, whereas epileptic dogs without obvious structural lesions or other known causes are categorized as having “idiopathic epilepsy.” Idiopathic epilepsy includes proven genetic epilepsy, suspected genetic epilepsy, and epilepsy of unknown cause (8). This classification scheme differs from the current classification in human epileptology, in which “idiopathic” has been replaced by “genetic” and “unknown etiology” (10).

Concerning classification by seizure semiology, the motor, sensory and behavioral characteristics of seizures, most dogs have generalized convulsive (tonic, clonic, or tonic-clonic) seizures. Generalized convulsive seizures can be primarily generalized or, more often, evolve after focal seizure onset (8). At least two-thirds of DWE exhibit focal-onset seizures with or without secondary generalization (8, 11). Focal seizures can be very subtle and may be difficult for a dog's owner to recognize. Focal seizures in humans may be characterized as an aura, which is simply a focal seizure producing a feeling that is without impairment of consciousness or function. Prodromal signs may precede a seizure, and generally are defined by their long-lasting nature (8).

A major problem in the correct diagnosis and classification of canine epilepsy is the lack of routine electroencephalographic (EEG) recordings in unsedated, behaving dogs (8). In human neurology, the scalp EEG is a key instrument in the epilepsy workup, guiding primary diagnosis, epilepsy classification, and treatment (7, 12).

**Figure 1:**
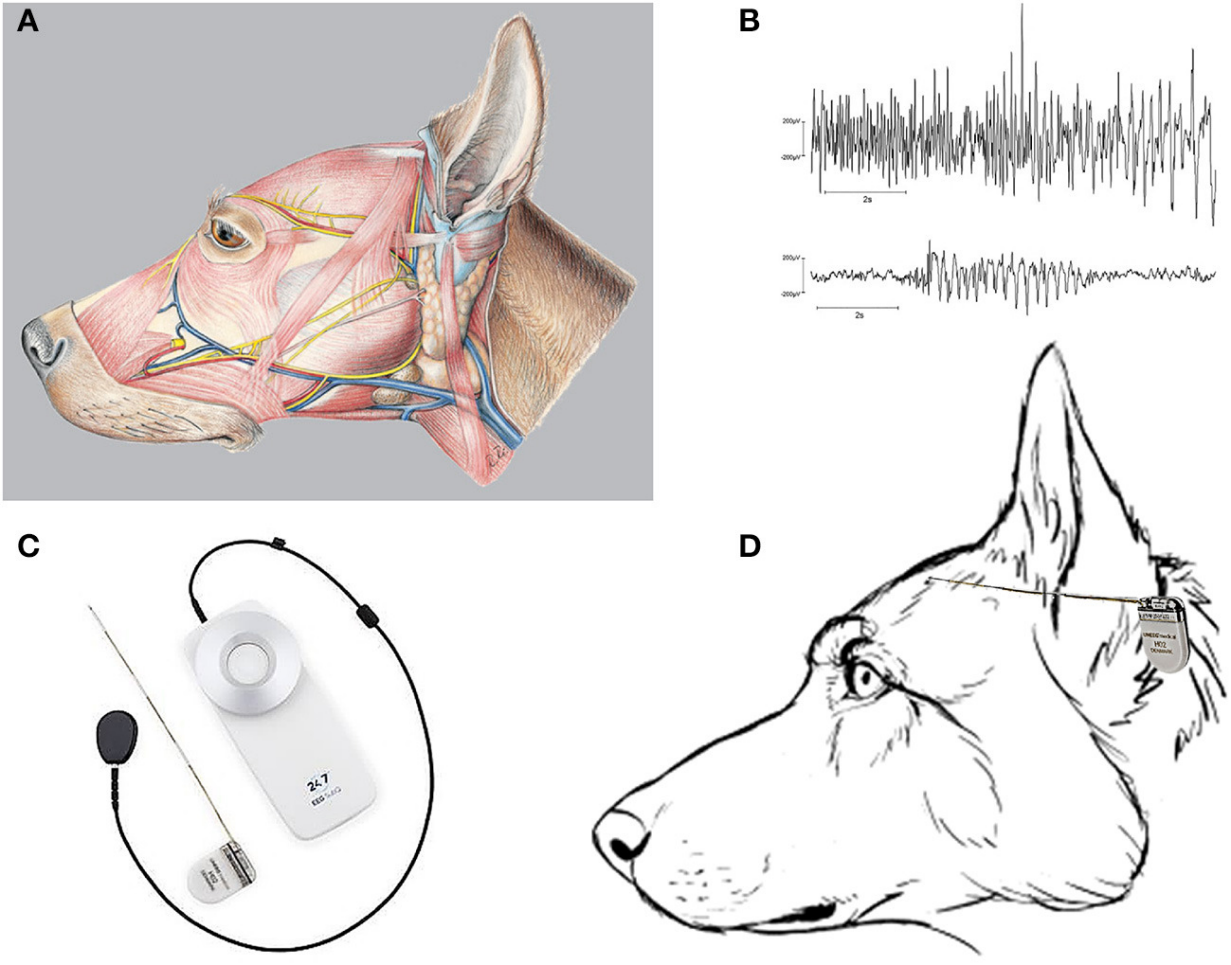
The musculature of the dog's head and seizure recording by a subscalp EEG device. **(A)** Illustrates the massive musculature of the dog's head (see also Figure 2D), which produces artifacts when applying scalp EEG electrodes. The picture was taken from Reese et al. (15). **(B)** Two different EEG seizures were recorded from Beagle dogs with subscalp electrodes inserted below the temporalis muscle on the skull to minimize electromyographic artifacts [from Authier et al. (16)]. EEG data were obtained using telemetry transmitter leads with bipolar derivations according to the internationally standardized 10–20 system, using Cz-Oz derivations as previously described (17). **(C)** The 24/7 EEG™ SubQ system from UNEEG Medical (Lillerød, Denmark), involving a small ceramic implant that consists of an electrode house containing an inductive coil for the transfer of power and data and a wire with three leads (electrodes). The center electrode is used for reference, and the recordings, therefore, have two bipolar channels. An external device supplies the implant with power, receives and stores the measured EEG signals, and when coupled with a smartphone can wirelessly stream the recorded EEG data automatically to a cloud environment. **(D)** Schematic illustration of how the 24/7 EEG™ SubQ system may fit to the size of a dog's head. The device will be tunneled under the temporalis muscle and proximate to the dog's skull (not illustrated; see text).

**Figure 2:**
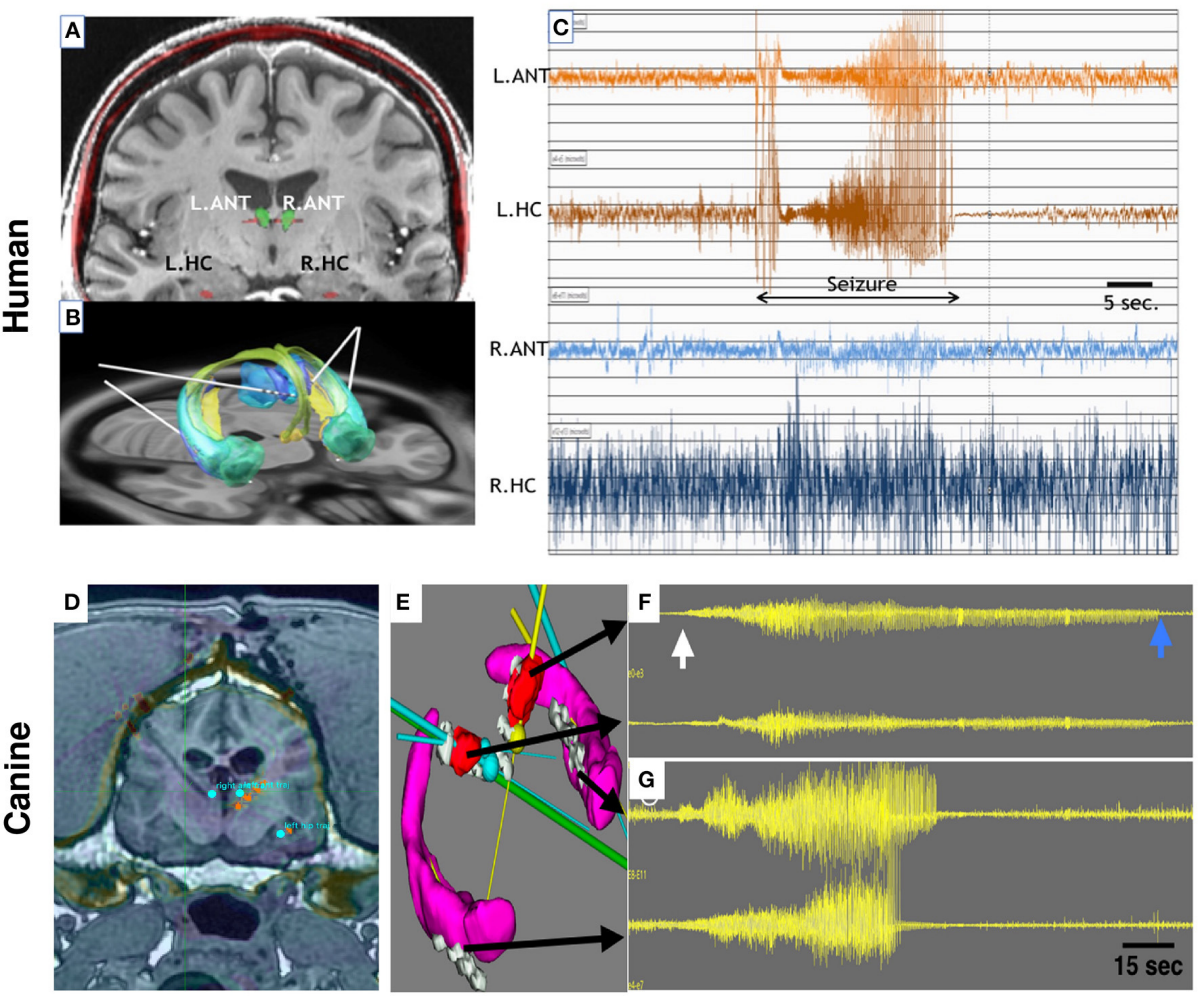
Bilateral Anterior Nucleus Thalamus (ANT) and Hippocampus (HPC) targets for mesial temporal lobe epilepsy in human **(A–C)** and canine **(D–G**) epilepsy. **(A)** 3T MRI of a human brain with targets for ANT(green) and HPC (red) electrodes labeled. **(B)** Papez circuit with ANT (blue) and HPC—AMG (green) targets and implanted electrode leads (white lines). **(C)** Spontaneous focal seizure with simultaneous left ANT and HPC involvement. Clinically the patient had intense fear and loss of awareness. The seizure has synchronous left ANT and HPC onset without the evolvement of contralateral ANT or HPC. **(D)** 3T MRI and stereotactic targeting in the canine brain. The bilateral ANT and HPC targets are highlighted. The coronal MRI shows the massive cranial musculature surrounding the canine skull. **(E)** Bilateral HPC (purple) and ANT (red) target volumes and implanted electrodes (gray). The multiple color straight lines indicate target trajectories for electrode leads. **(F,G)** Four channels of intracranial EEG. From top to bottom: Left ANT, Right ANT, Left HPC, and Right HPC recordings. The onset of a seizure (white arrow) and the seizure offset (blue arrow) are clearly evident in both HPC and ANT recordings, but interestingly the seizure terminates in all electrodes at different times. The longest seizure discharge is in the left ANT (top trace). See text for details. ANT, anterior nucleus of the thalamus; HPC, hippocampus. Adapted from Sladky et al. (18).

The EEG can also be used to detect abnormal interictal (between seizures) epileptiform activity and plays a crucial role in the pre-surgical evaluation of people undergoing epilepsy surgery (13, 14). Interictal epileptiform discharges are brief, less than a second, EEG transients not associated with symptoms or signs that are unique to epilepsy. In contrast to humans, because of EEG artifacts caused by the dog's heavy muscles on the skull (Figures 1A, 2D), non-invasive scalp EEG recording has never been established as a standard laboratory technique for the diagnosis of canine epilepsy (19). In some neurological referral hospitals, subdermal needle scalp electrodes have been employed to solve this issue, although doing so requires heavy sedation or anesthesia, which may impact interictal and ictal EEG recordings on the dog (20). To lessen this issue, ambulatory EEG recording may continue after the patient has recovered to a normal state of consciousness when sedation or general anesthesia was used for the implantation of EEG electrodes (21); however, this has only been used for relatively short EEG recording periods and remains technically challenging. Furthermore, in contrast to humans, there is no standardized recording method for EEGs (22) in dogs and thus no specific consideration of electrode arrangement, montage, or immobilization.

There have been numerous reports of EEG recordings in epileptic dogs, but most of these reports had low detection rates of EEG abnormalities most likely because of the sedation or anesthetic utilized, duration of recording, and challenges of artifacts (23). Furthermore, as in people, the vast majority of EEG recordings in dogs are performed in hospital or practice settings and are of brief duration. Unless new implantable EEG devices and methods of continuous (24/7) EEG monitoring become available in unsedated, behaving dogs, it is doubtful that EEG recordings in DWE can be used to characterize seizures. In this review, we will describe and discuss novel subscalp and intracranial devices to wirelessly record and analyze continuous (24/7) EEG in unsedated dogs. Although these devices have been developed for humans, dogs are large enough to accommodate such devices (3). Importantly, continuous intracranial EEG (iEEG) recordings in unsedated epileptic dogs were indistinguishable from human EEG recordings, both in terms of ictal and interictal EEG alterations (2, 24, 25), further substantiating the similarities between canine and human epilepsy (examples of EEG seizures in dogs are shown in Figures 1B, 2F,G, highlighting the similarity of canine and human recorded seizures). Thus, we envision that continuous EEG monitoring in DWE will provide a new paradigm in the diagnosis and prognosis of canine epilepsy, particularly for dogs with infrequent seizures. Some of the available EEG devices discussed here can be used for effective detection of seizures and caregiver alert (26) and also provide better than chance seizure forecasting minutes to hours before a seizure occurs (27). Furthermore, continuous EEG monitoring would allow accurate detection of temporal seizure patterns, seizure cycles, and seizure frequency, and likely improve medication optimization. In addition, and again similar to human epilepsy this technology should help resolve the low accuracy of seizure diaries compiled by owners of DWE.

In the following, we describe multiple systems enabling long-term EEG recordings. Here we focus on systems that have been tested in pre-clinical and clinical studies. The cost and regulatory challenges of developing, testing, and validating a fully implantable EEG acquisition system are considerable (28). It is common for new devices and companies to not achieve a commercial release. The examples below are not meant to be exhaustive, but more to highlight companies and devices with significant published research demonstrating viable system development. The regulatory and development barriers are higher for humans compared to canines, and this further highlights the opportunity for parallel development in canines and humans as was demonstrated with the NeuroVista device (24, 27, 29–31).

## Subscalp devices for long-term EEG recordings

In 2017, James et al. (32) reported the outcome of a proof-of-concept trial on the diagnostic utility of wireless video-EEG in unsedated dogs with presumed seizures. In the study, subscalp (subdermal) wire electrodes or needle electrodes were used, and they were attached to the scalp using a sticky bandage, either with or without a separate non-adhesive bandage to secure the leads. Electrode placement expanded upon a previously described protocol with 8 EEG channels (33), adding up to five more electrodes to increase coverage of the cortex. Routine electrodes included reference, ground, midline, frontal, central, temporal, and occipital electrodes. The median EEG recording duration was 1.5 h (range: 0.17–22.5 h). Eighty-one dogs were enrolled in the study. The clinical question for all dogs undergoing video-EEG was whether the abnormal behavioral events observed in these dogs are epileptic seizures. This is an important and not trivial question because, for instance, canine paroxysmal dyskinesias may be misdiagnosed as seizures without EEG (34). In the study of James et al. (32), EEG analysis achieved/excluded a diagnosis of epilepsy in 58 dogs (72%); 25 dogs confirmed with epilepsy based on recording ictal or interictal epileptiform discharges, and 33 dogs with no EEG abnormalities associated with their target events. James et al. (32) concluded that wireless video-EEG in unsedated dogs had high success in the diagnosis of unusual behavioral events. However, the main restriction of this study was the short duration of EEG monitoring. Epilepsy cannot be reliably excluded by EEG monitoring for only up to 24 h. Furthermore, in the study of James et al. (32), video-EEG monitoring was done in the veterinary clinic and not in the dogs' natural environment. These shortcomings can be partially resolved by implantable EEG devices that provide continuous recordings over several months or more.

Several studies in human subjects have helped clarify other aspects of subscalp EEG recordings (35). For instance, Bacher et al. (36) recorded awake and ictal EEG epochs obtained from 21 human epilepsy patients with subscalp electrodes and validated them against simultaneous iEEG recordings. The subscalp electrodes were placed subgaleally (just under the scalp above the skull). For each patient in the data collection, a subject-specific seizure detection algorithm was developed and then offline assessed, resulting in 97% sensitivity, 91% specificity, and 93% accuracy (36). The researchers concluded that a straightforward seizure detection algorithm using subscalp EEG signals could provide adequate clinical value and accuracy for a long-term, low-power subscalp brain monitoring system. The same group reported that single-channel subgaleal EEG, placed at or near the vertex, accurately identified 98% of iEEG-verified temporal and extratemporal onset seizures with a sensitivity of 98% and specificity of 99% (37).

Do Valle et al. (38) described the design of a subscalp eight-channel EEG recorder and seizure detector that has two modes of operation, diagnosis and seizure counting, with electrode arrays projecting cranially in a fanlike pattern from behind the ear of humans. The authors suggested that their device has advantages vs. ambulatory scalp EEG systems, which only last up to 3 days and are thus far from ideal for patients that have infrequent seizures, and iEEG solutions that require a craniotomy. However, as discussed in the next section, in recent years long-term iEEG recordings have proved useful to localize the origin of seizures, studying temporal seizure patterns and seizure frequencies, and developing algorithms for seizure forecasting. The epileptic dog has been a valuable translational animal model for long-term iEEG.

Several devices for recording subscalp EEG have been developed in recent years (Table 1) (35). One of these, the 24/7 EEG™ SubQ system from UNEEG Medical (Lillerød, Denmark) has been approved for use in humans in the EU and is undergoing evaluation and investigational trial in the US (Figure 1C). This device appears to be particularly well-suited for dog studies. However, to our knowledge, none of the subscalp EEG systems described in the following has been used in dogs, yet.

**Table 1:** Overview and characteristics of known subscalp EEG devices certified or currently in development for humans.

**Company**	**Device**	**Channels/montage**	**EEG sampling rate**	**Battery**	**Wearable companion**	**Continuous raw data available**	**Status**
UNEEG medical	24/7 EEG™ SubQ	2 channels/unilateral (but implantation of 2 devices for bilateral recording possible)	207 Hz	External/24 h rechargeable	Yes; the small external device receives and stores the measured EEG signals and is capable of 30+ days storage of EEG data	Yes; the implanted needle electrode can be used for up to 15 months	European CE approval in 2019; FDA approval pending
Epi-Minder	Minder™	2 channels/bilateral	250 Hz	External/24 h rechargeable	Yes	Yes	Clinical trials ongoing
Wyss Center	Epios™	7 channels/temporal OR 14 channels/bitemporal OR 28 channels/full montage	250 Hz	External/24 h rechargeable	Yes	Yes	Clinical trial underway
BrainCare Oy	UltimateEEG™	1 or 2 channels/unilateral up to 8 channels/unilateral	256 Hz	Internal/1 year; external/24 h rechargeable	Packaged into a unit behind the ear	No; only relevant epochs	Clinical trial announced
Neuroview Technology		1 or 2 channels/unilateral	256 Hz	Internal/1 year	No	No; only relevant epochs	Clinical trial announced

Based on Duun-Henriksen et al. (35) and data provided by the manufacturers. “?” indicates that data are not yet disclosed. To our knowledge, none of these devices has been used in epileptic dogs, yet.

The three subscalp EEG systems discussed in more detail below (24/7 EEG SubQ system, Minder, and Epios) have at their core a technology refined over decades for hearing restoration (39). Both UNEEG and Epiminder have at their beginnings connection with companies making cochlear implants. Similar to modern cochlear implants, these EEG systems use small implantable devices that are externally powered through a separate inductively coupled external device. The elimination of an implanted battery to power the device, memory to store data, and a microprocessor to analyze data massively reduce the complexity and size of the implanted device. The EEG data are only recorded, to an external memory, when the implant is powered by the external power source that is inductively coupled through the skin to the implant. All data storage and analysis occurs on an external device, such as a smartphone, cloud environment, or other bespoke external hardware systems. This massively reduces the implant cost and size. A primary limitation, however, is that EEG data are only recorded if the external power supply is connected, usually through a small wire tether for interfacing the external device to the implanted device (e.g., Figure 1C).

### The 24/7 EEG™ SubQ system from UNEEG medical (https://www.uneeg.com/)

In people, this subscalp device, a small ceramic implant that features two bipolar channels (Figure 1C), is inserted with a needle inducer under local anesthesia. A small external device is inductively coupled to the fully implanted acquisition system. The external device receives and stores the measured digitized EEG signals and is capable of 30+ days of storage of EEG data (2 channels, 207 Hz sampling, 10 bit). The external device supplies the implant with power through the inductive link (wireless). The recorded EEG data can be streamed to a secure cloud environment for storage, analysis, and visual review. The EEG data are analyzed by automated seizure detection algorithms, and suspected seizure activity is highlighted for subsequent expert visual review.

The SubQ device was used to record EEG in healthy human subjects (40) as well as to detect clinically relevant EEG seizures in people with epilepsy (PWE), and demonstrates high reliability and good patient tolerance (41–44). Visual confirmation of the accuracy of the sub-scalp system's seizure identification was provided by periods of concurrent conventional scalp EEG data (45). In a case study in which the EEG device was used to provide an objective seizure count, the SubQ device identified unrecognized breakthrough seizures and informed medication treatment response, prompting anti-seizure medication (ASM) adjustment (46).

The SubQ device is integrated with software for automatic seizure detection, EEG visualization, and annotation. The device is CE-marked, and multiple clinical trials are currently ongoing. The implant is approved for a lifetime of ~1.5 years, thus allowing ultra long-term EEG monitoring in canines and humans living in their natural environments. The data acquired by this system also allowed seizure forecasting to be successfully undertaken (44, 45, 47). The area under the receiver operating characteristic curve (ROC AUC score) achieved 0.88, which is comparable to the performance of state-of-the-art forecasting work using iEEG (27, 45). The primary limitation of seizure forecasting has remained the modest specificity with as high as 30% time in warning required to achieve high AUC.

Patient diaries are frequently used in longitudinal epilepsy therapy, although they are notoriously unreliable for many PWE, particularly those who have focal impaired awareness or nocturnal seizures (48, 49). Thus, patients with subtle or nocturnal seizures may benefit most from the new UNEEG subscalp device. As in people, seizure counts in DWE guide individual treatment decisions and are often the primary outcome measure of pharmacological trials. Seizure diaries have sensitivities in the range of only 30–50% (21, 48). Thus, as in people (48, 50), objective seizure monitoring in DWE would not only lead to a better diagnosis of the type of epilepsy and seizures but also improve pharmacologic treatment and optimization. Indeed, the unpredictability of seizures is a crucial factor in the management of canine epilepsy, and dog owners have a great desire to know when a seizure happens, according to a recent survey of owners of epileptic dogs (51). According to the study by Bongers et al. (51) on dog owners' perceptions of seizure detection devices, owners thought these devices would help them better manage their dog's seizures, including by providing more accurate seizure frequency monitoring and making it easier to administer emergency medications when necessary. The seizure monitoring system that was preferred by dog owners was one that used a seizure detection device, and a wearable device was recommended above an implant. There are numerous dog health collars available right now, but none has shown to be a reliable seizure detector. In a study by Munana et al. (52) on a collar-mounted accelerometer in DWE, generalized seizures in dogs could be detected, but the overall sensitivity was low. In another study on a wearable automatic seizure detection system using acceleration data and the Mahalanobis distance in DWE with generalized tonic-clonic seizures (GTCS), the GTCS-detecting algorithm created for this study was effective in identifying all acceleration data of GTCSs as seizures and all acceleration data of daily activities as non-seizure activities (53). However, the sample size was low and focal seizures were not detected, which is a main disadvantage of wearables.

We expect that subscalp systems such as the UNEEG device would be much more sensitive than wearables to detect both focal and generalized seizures in dogs, again similar to what has been found in PWE (54). As shown in Figure 1D, the UNEEG SubQ subcutaneous implant (which will be tunneled under the temporalis muscle and proximate to the dog's skull) may fit to the skull of a larger dog breed, which we are currently evaluating in cooperation with UNEEG and the group of Holger A. Volk (Department of Small Animal Medicine and Surgery, University of Veterinary Medicine) in Hannover. Implantation of two devices would allow EEG recording from both hemispheres. Furthermore, in contrast to humans where strict rules prevent the reuse of implantable devices, the devices can be repeatedly sterilized and reused in different canine patients, thus significantly reducing the overall costs.

Interestingly, in a recent study on people with temporal lobe epilepsy, the subcutaneous EEG was combined with an electromyographic (EMG) estimate and chest-mounted accelerometry (55). This allowed calculating multimodal ictal fingerprints that characterized the seizures of each patient. Furthermore, home video combined with ambulatory EEG has demonstrated clinical utility (12, 56). Such multimodal methods would be highly interesting, and likely useful for canine epilepsy.

### The Minder™ device from Epi-Minder (https://epiminder.com/)

A multichannel electrode lead implanted across the skull with a tunneling technique is how the subscalp device known as Minder from Epi-Minder (Melbourne, Australia) obtains subscalp EEG from both hemispheres (35, 45). The fully implanted EEG acquisition device digitizes subscalp EEG captured from the implanted electrode and - similar to the UNEEG and Epibios devices - transmits these signals to a wearable data storage device that is inductively coupled to the fully implanted device and provides power to the implant and Bluetooth connection to a smartphone. A Minder companion application running on a smartphone is used to collect EEG data for analysis and review. The EEG is wirelessly streamed from the smartphone to a cloud environment for long-term storage, analysis, and review. The Minder platform has the potential to provide long-term, continuous quantitative measures of the subscalp EEG to support improved diagnosis and management of epilepsy. In Australia, a prospective clinical trial is now being conducted in PWE to evaluate the safety of the subscalp monitoring device for the recording of brain electrical activity related to the incidence of epileptic seizures.

### The Epios™ device from the Wyss Center (https://wysscenter.ch/advances/epios-brain-monitoring)

The Wyss Center for Bio and Neuroengineering's Epios™ system, which was developed in Geneva, Switzerland, aims to provide flexible configurations, from focal or bitemporal electrode layouts to provide broad coverage by transposing the locations of the full 10–20 scalp EEG montage to the subscalp compartment (35). Under general anesthesia, the entire montage is implanted in people through two to four small (1 cm) incisions in about an hour using specialized epiosteal tunneling instruments. With lower coverage, implantation under mild sedation or local anesthesia may be possible. The wireless subscalp EEG signals are sent to a receiver that is held in place on the skin directly over the implant with a magnet, and to a custom wearable data storage and analysis processor and are finally securely stored in the Epios Cloud. For multimodal coregistration, the Epios wearable has a three-axis accelerometer, audio recorders, and a heart rate monitor. All data are transmitted to a secure cloud-based application developed to support long-term data visualization and analysis. Modern event detection algorithms that can automatically identify and present regions of interest to augment clinicians' visual screening in applications like epilepsy are included in the cloud software. It is designed to be used as a medical device, giving authorized medical professionals remote access to and analysis of the data. Following the successful termination of preclinical studies, clinical trials are now underway with the Epios early feasibility study (https://www.clinicaltrials.gov/ct2/show/NCT04796597) in collaboration with the sleep-wake epilepsy center at the University Hospital of Bern (INSELSPITAL; Bern, Switzerland).

### The Epicranial application of stimulation electrodes for epilepsy (EASEE^®^) device from Precisis

A particularly novel application of subscalp electrophysiology is the EASEE^®^ device from Precisis (Heidelberg, Germany). The EASEE^®^ device uses a novel five subscalp platelet electrode configuration (four smaller electrodes arranged around a larger center one) (35). The surface Laplacian notion served as the basis for this design, which improves stimulation depth. It can record and give neurostimulation in a customized closed-loop environment, and it is intended to be implanted above a lesioned brain region and/or epileptogenic focus. PWE are guaranteed complete freedom of movement thanks to the thin platelet electrodes, which are invisible from the outside. Two clinical investigations demonstrating the high efficacy of EASEE in PWE were conducted in various European epileptic centers under the direction of Professor Schulze-Bonhage (Freiburg, Germany) (57). The product is expected to be launched in Europe in 2022. In February 2022, Precisis received the Food and Drug Administration (FDA) breakthrough device designation for the minimally invasive EASEE device.

There are multiple other devices (see Table 1) (35) under development for PWE, but limited published data currently to evaluate their performance. Two of these devices are shortly reviewed in the following.

### The UltimateEEG™ device from BrainCare

Platinum on silicon electrodes with custom order sizes, number of channels, and electrode spacing are used in UltimateEEG by BrainCare Oy (Tampere, Finland). The patented electrode technology enables customizable electrodes to capture seizure propagation with low noise recordings due to the flexible, planar design. The electrodes are available as 4–8 channels per strip and the electrode strips can be connected for a total of 4–16 channels. The motivation is to go beyond seizure counting, which is only possible with 2 channels, allowing to measure seizure propagation, seizure strength/intensity, and a very limited local network over the focal area. The design of the electrodes allows to place them over any of the major lobes subdermally (frontal, occipital, parietal, and temporal). The raw data will be available to clinicians and anonymized/de-identifiable data can be accessible to researchers. The sampling rate of the current hardware is 1,000 Hz. Battery life is 1–2 days, depending on the number of channels. The external wearable is designed as small as possible and packaged into a behind-the-ear unit. A clinical trial has been announced.

### The device from Neuroview Technology

The Englewood, New Jersey, USA-based company Neuroview Technology Ally is developing a fully implantable subscalp EEG recording technology to help diagnose and quantify seizures in PWE (35). The implanted device can record the EEG continuously for a year without needing to be recharged. Epochs of subscalp EEG activity suspicious for seizures and patient-identified events are recognized by low-power, onboard algorithms. With the use of cloud-based machine learning algorithms, EEG epochs are sent to a cloud platform *via* a connected smartphone application for the neurologist to analyze and confirm seizures and display and quantify seizure activity in between clinic visits. The on-device detection algorithms can be altered to increase seizure detection's specificity. Clinical trials are announced.

## Intracranial devices for long-term EEG recordings

Devices for long-term iEEG recording can be subdivided into implantable neural sensing and stimulation (INSS) devices, which both record the intracranial EEG and deliver electrical brain stimulation, and sensing-only devices (Table 2). There is not currently an approved clinical ambulatory invasive iEEG sensing-only device.

**Table 2:** Overview and characteristics of intracranial EEG (iEEG) devices certified or currently in development for humans.

**Company**	**Device**	**Number of leads/contacts**	**ECoG sensing (N channels from N contacts)**	**Sample frequency (Hz)**	**Stimulation Modes** **(A) Continuous (B) Duty cycle** **(C) Responsive** **(D) Programmable adaptive**	**Rechargeable**	**External companion**	**ECoG storage on device**	**Evaluated in dogs with epilepsy**	**Current status**
NeuroPace	RNS^®^ system (for recording and stimulation)	2/8	4/8	250	(A) No (B) No (C) Yes (D) No	No	Yes	3,600 s	Yes	Approved for use in humans in 2013
Medtronic	Percept™ (for recording and stimulation)	2/16	2/16	250	(A) Yes (B) Yes (C) No (D) No	No	Yes	No LFP data	No	Approved for use in humans in 2020
Medtronic	RC+S (for recording and stimulation)	4/16	4/16	250–1,000	(A) Yes (B) Yes (C) Yes (D) Yes	Yes	Yes	None	Yes	Investigational device; will sunset 2022
NeuroVista	SAS	4/16	16/16	400	(A) N/A (B) N/A (C) N/A (D) N/A	Yes	Yes	1 week	Yes	Dissolved SAS not available
CorTec	Brain Interchange One	2/32	32/32	1,000	(A) Yes (B) Yes (C) No (D) No	No (requires inductive connection)	Yes	None	Yes	Investigational

Based on the literature discussed in the text and data provided by the manufacturers. N/A, not available.

There are two clinically available epilepsy INSS devices, the Neuropace Inc. responsive neurostimulator (RNS^®^) and the Medtronic Inc. Percept™ deep brain stimulator. The RNS device continuously records iEEG and device-embedded algorithms trigger electrical stimulation to reduce seizures in PWE. The Percept deep brain stimulation (DBS) provides continuous streaming of iEEG in the clinic and averages power-in-band time-series chronically in PWE (58). The use of RNS targeting epileptogenic networks (59) and duty cycle DBS of the anterior nucleus of the thalamus (ANT) (60) in PWE have proven effective in reducing patient-reported seizures, but both devices have limited memory for storing iEEG data and do not provide accurate seizure diaries.

### The NeuroPace RNS^®^ system

The NeuroPace RNS^®^ system (Mountain View, CA, USA), the first device to provide closed-loop brain-responsive neurostimulation in PWE, was approved by the FDA in 2013 as adjunctive therapy for adults with medically refractory focal-onset epilepsy with 1–2 seizure foci (Table 2). The system continuously records the iEEG and embedded algorithms are trained to recognize and respond to each patient's unique brain patterns of seizures and interictal activity, providing personalized stimulation and preventing seizures before they start (61). The RNS system consists of two four-electrode cortical strips and/or depth leads put intracranially at the epileptogenic focus/foci, as well as a cranially implanted neurostimulator that houses the electronics and a primary cell battery (62). This system provides continuous sensing of neural activity, and in response to detections of abnormal (i.e., epileptiform) activity, it delivers electrical pulses intended to terminate incipient seizures. Clinical trials showed that brain-responsive neurostimulation is acceptably safe, reduces seizure frequency, and improves the quality of life in adults with medically refractory focal-onset epilepsy (59, 61). Whereas, the total volume of resective epilepsy surgeries decreased in recent years, RNS implantations have increased by over 100% in persons with drug-resistant epilepsy (63).

### The Medtronic Percept^®^ DBS system

DBS of the ANT has a CE mark and US FDA approval for the treatment of focal DRE in PWE [SANTE trial (60, 64)], and the recently approved Percept device provides sensing from the ANT (Table 2). The commercially available Medtronic Percept DBS device provides streaming of iEEG in the office and chronic long-term local field potential (LFP) power data within a physician-specified frequency band saved in 10-min averaged increments. There is some evidence for the use of these LFP power-in-band (PIB) trends for tracking epilepsy (58).

To our knowledge, the NeuroPace RNS^®^ and Medtronic Percept systems have not yet been used in epileptic dogs, but an investigational device from Medtronic (RC+S™), which is capable of continuous iEEG data streaming and adaptive electrical stimulation, has been employed in DWE.

### The Medtronic RC+S™ summit system

**Figure 3:**
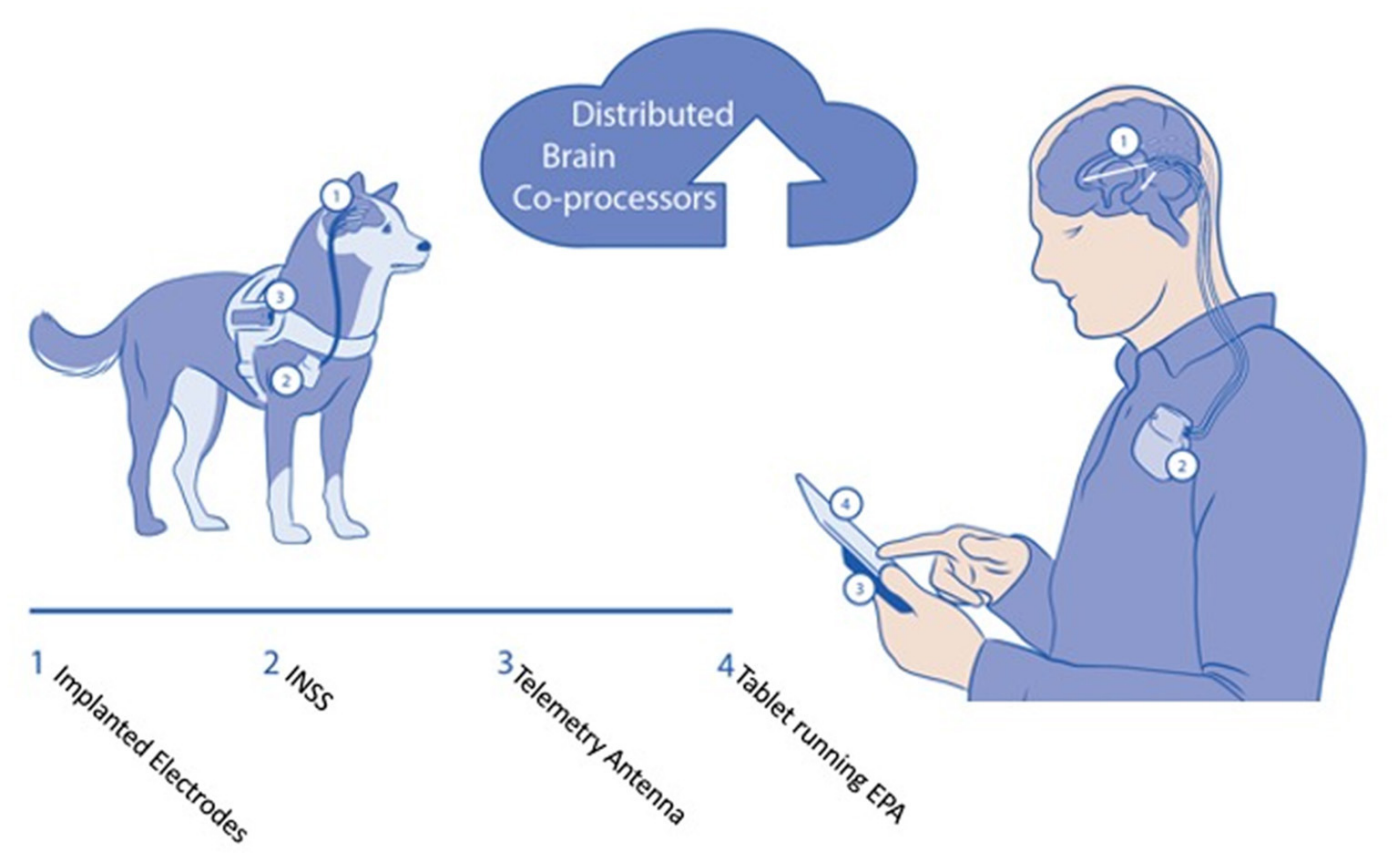
Schematic of brain co-processor system for canines and human epilepsy. The system consists of: (1) Implanted lead with multiple recording and stimulation contacts (2). Implanted neural sensing and stimulation device. In the canine, the INSS is implanted adjacent to the scapula, and in humans below the clavicle (3). The embodiment of the system using the Medtronic RC+S™ Summit device requires a telemetry antenna (4). The tablet and smartphone can run the epilepsy patient assistant application that integrates the hardware device and provides bi-directional connectivity to the cloud environment. Automated algorithms for detecting seizures and interictal activity run continuously in the cloud and tablet devices.

Medtronic Inc. (Minneapolis, MN, USA) recently designed a novel experimental device with EEG telemetry and electrical therapy modulation capabilities (65). The investigational Medtronic Summit RC+S™ system (Figure 3) was developed to telemeter iEEG in PWE, provide on-device seizure detections, and modulate stimulation therapy based on either on-board EEG analytics or analytics from a smartphone or mobile computer (Table 2). In contrast to prior chronic brain recording studies with the NeuroPace RNS system with constrained clinician-defined detectors, the RC + S™ device is uniquely suited to evaluate the longitudinal impact of neuromodulation on cycles in epilepsy (25).

In a first feasibility study in dogs, Kremen et al. (66) demonstrated the feasibility of adaptive brain stimulation, continuous sensing, and embedded and off-the-body analytics. Continuous iEEG telemetry from the RC+S device to a handheld or tablet computer provided advanced analytical capability. A total of seven dogs were implanted, four dogs with naturally occurring epilepsy and three controls, and an average of 293 days of recording were collected per animal to test the seizure detection performance of the device. Furthermore, stimulation was performed for 48 h per dog in four subjects, demonstrating that more than 97% of telemetered data was fully analyzable (66). The ability to track behavioral states (67) and create accurate seizure diaries (18) during electrical brain stimulation has been demonstrated in both dogs and humans.

In a subsequent canine study, Gregg et al. (68) used the Medtronic Summit System RC+S and the NeuroVista SAS (see below) for characterizing circadian and multiday seizure periodicities, and seizure clusters in dogs with naturally occurring focal epilepsy. Furthermore, relationships between inter-seizure interval and seizure duration were evaluated. The study showed that seizure timing in epileptic dogs is not random and that circadian and multiday seizure periodicities and seizure clusters are common, similar to recent reports in humans (69). Circadian, 7-day, and monthly seizure periodicities occur independent of ASM dosing, and these patterns likely reflect endogenous rhythms of seizure risk (68, 70, 71). To our knowledge, this was the first study to objectively characterize circadian and multiday seizure periodicities and seizure clusters in dogs with naturally occurring epilepsy. The study demonstrates the potential usefulness of long-term continuous (24/7) seizure monitoring for characterizing canine epilepsy.

Based on iEEG monitoring, electronic seizure diaries, and preclinical experiments in various species, including dogs and humans, it is now clear that seizures do not occur at random but at cycles that operate over diverse timescales: daily (circadian), multi-day (multidien), and yearly (circannual or seasonal) (70). The mechanisms underlying these cycles are intensively investigated (70, 72). Seizure cycles are not driven mainly by medication but several animal studies, including studies in dogs (see above), have established the existence of multiday cycles of epileptic activity in the absence of ASMs (71). However, the circadian rhythm of seizure occurrence may be influenced by ASMs (71). Furthermore, the therapeutic effect of ASMs may be affected by chronobiological rhythms. For instance, we found a circadian regulation of ASM targets that was affected by experimental temporal lobe epilepsy in mice (73). Furthermore, we found striking seasonal alterations in ASM efficacy in rodent models of epilepsy (74). If these findings can be translated to patients, such alterations need to be considered when designing drug treatments and timing their delivery.

**Figure 4:**
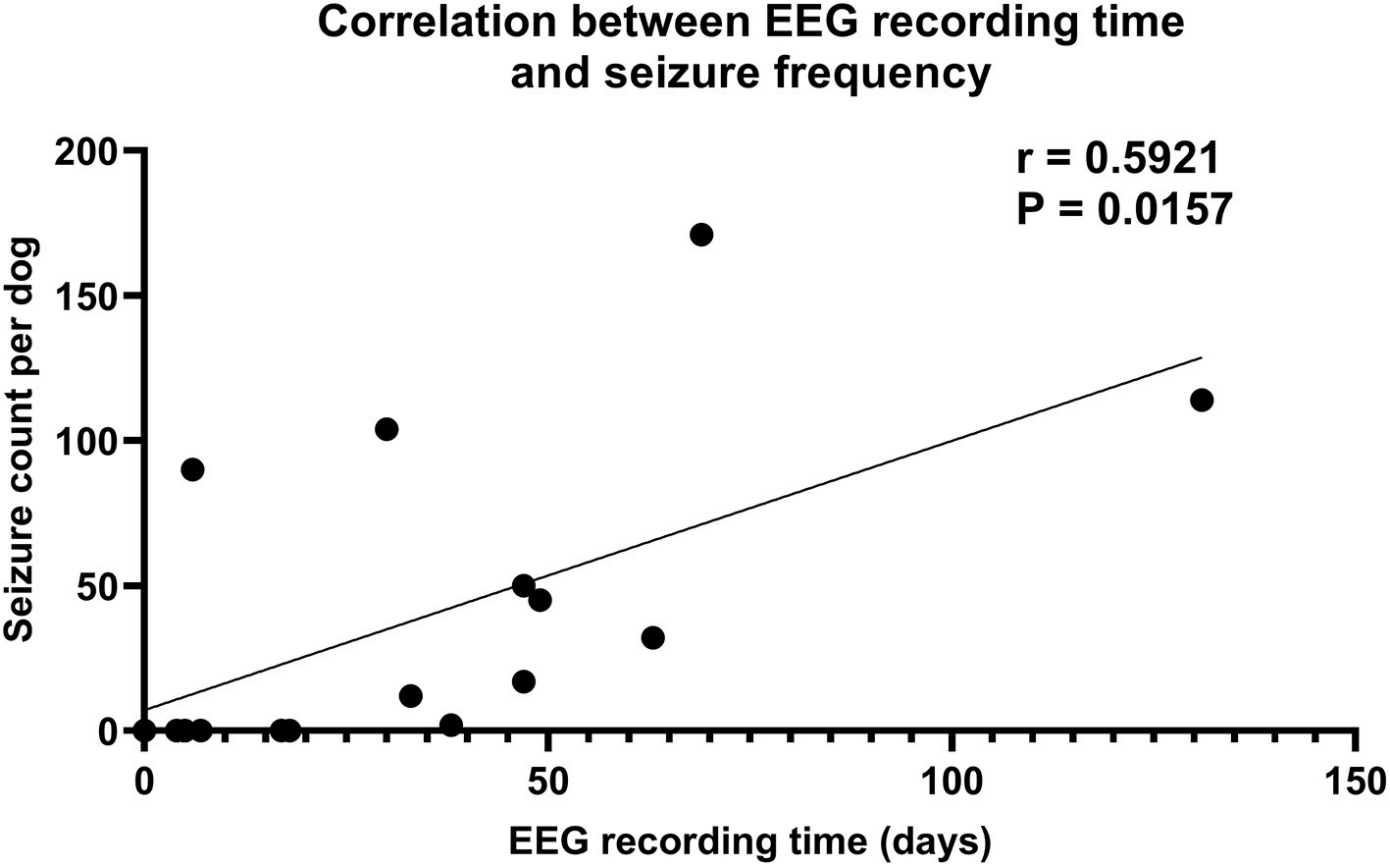
Correlation between intracranial EEG (iEEG) recording time and seizure frequency in 16 dogs with naturally occurring epilepsy. For continuous (24/7) ambulatory iEEG recordings, either the NeuroVista Seizure Advisory System (SAS) or the Medtronic Summit System RC+S were used. The data show that the longer the recording time, the higher the chance of detecting spontaneous seizures in epileptic dogs. Data are from Gregg et al. (68).

An additional aspect shown by the dog study of Gregg et al. (68) is the impact of the EEG recording time on seizure determination. In the 16 epileptic dogs used, seizures were detected by continuous EEG monitoring in 10 (63%). The average duration of EEG monitoring in these 10 dogs was 51.3 ± 10.5 days compared to 8.5 ± 2.9 days in dogs without detected seizures (*P* = 0.0081). In addition, there was a significant positive correlation between the duration of EEG monitoring and seizure frequency in the 16 dogs (Figure 4), again demonstrating that—because of the periodicity of spontaneous seizures—the chance of detecting seizures increases with the duration of EEG monitoring. This is substantiated by the low seizure detection rates of ambulatory EEG monitoring previously used in dogs (21, 75), underlining the usefulness of long-term EEG monitoring in the diagnosis of canine epilepsy.

In a subsequent study by the same groups (25), again using the Medtronic Summit System RC+S and the NeuroVista SAS devices in seven epileptic dogs and one human with epilepsy, circadian and multiday cycles in the rate of interictal epileptiform spikes (IES) were observed. There was seizure phase-locking to circadian and multiday IES cycles in five and seven out of eight subjects, respectively. Two pet dogs and the human subject received concurrent DBS of the ANT over multiple months (Figure 2). Thalamic DBS modified circadian (all 3 subjects) and multiday (analysis limited to the human participant) IES cycles. The authors concluded that multiscale cycles in brain excitability and seizure risk are features of human and canine epilepsy and are modifiable by thalamic DBS (25). In humans, the effectiveness of bilateral stimulation of the ANT has been well-established in a multi-centers randomized trial [SANTE trial (60, 64)], and ANT DBS has a CE mark and FDA approval for the treatment of drug-resistant focal epilepsy. Interestingly, in a dog with drug-resistant epilepsy, DBS of the centromedian nucleus of the thalamus prevented cluster seizures and status epilepticus (76).

### The NeuroVista SAS device

One of the major advances in human clinical epileptology was the development of the NeuroVista seizure advisory systems (SAS) that was used for long-term EEG recordings and seizure forecasting in PWE (29–31, 62) (Table 2). The goal of the investigational NeuroVista SAS device (Seattle, WA, USA) was to develop an implantable EEG-based brain monitoring and seizure forecasting system (28). The creation of this technology involved several steps: (i) the creation of a sizable, high-quality database of EEG recordings; (ii) the application of a structured approach to algorithm development; (iii) the design and construction of an implantable 16-channel subdural neural monitoring and seizure advisory system; (iv) preclinical research using a canine model; and (v) a first clinical study of seizure forecasting in 15 in PWE followed for 2 years was conducted. By now, numerous studies on both dogs and people with epilepsy have been performed. The epileptic dog was extremely useful to provide a rich iEEG dataset of unprecedented length for studying seizure periodicities and developing new methods for seizure forecasting (77–79).

**Figure 5:**
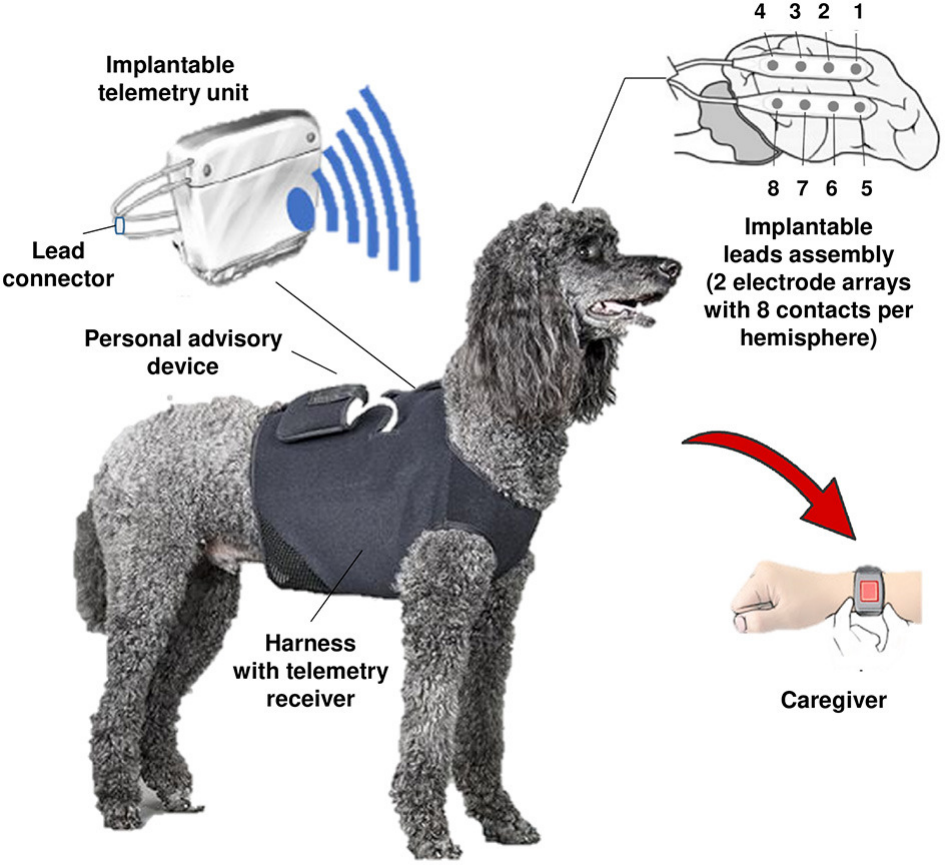
Schematic of a dog with an implanted ambulatory NeuroVista Seizure Advisory System (SAS). The implantable device for recording and storing continuous iEEG includes: An Implantable Lead Assembly (ILA) placed in the subdural space, an Implantable Telemetry Unit (ITU), and a Personal Advisory Device (PAD). The ILA, which acquires 16 channels of iEEG, detects and relays electrical activity in the brain to the ITU. The ITU receives data from the implantable leads, predicts seizure activity using an algorithm, and sends a wireless alert to the PAD. The PAD sends a wireless alert to the caregiver, which may lead to accelerated intervention and administration of seizure-stopping medication (see text). All iEEG data are stored on a flash drive and uploaded weekly *via* the internet to a central data storage site. Modified from Coles et al. (80).

In 2011, the first dog study was reported by Davis et al. (24) who tested the NeuroVista SAS device in six unsedated epileptic dogs over 5 months. Two electrode arrays with 16 intracranial sensors each were inserted into the subdural space for iEEG monitoring to record the iEEG from both hemispheres (Figure 5). The intracranial sensors were connected to a subclavicular acquisition and transmission unit that was implanted, rechargeable, and continually transmitted iEEG data to an external processing unit for real-time data storage, analysis, and communication of analysis results to caretakers. On real-time canine iEEG, a seizure detection algorithm was applied that has been trained on human iEEG data (24). In these animals, Davis et al. (24) showed previously uncharacterized intracranial seizure onset patterns that remarkably resemble human focal onset epilepsy. Subsequently, this device was employed to mine continuous iEEG in focal canine epilepsy, and for seizure detection and warning (26) and forecasting (27, 30, 78, 81).

Investigating interictal bursts and their electrographic connection to seizures was one of the objectives of these investigations. Focusing on the challenges of novel devices that continually monitor and analyze human EEG data over extended periods was another objective. Ung et al. (78) discovered significant temporal variability in seizures and interictal bursts following electrode implantation that took several weeks to establish a steady state in a subsequent year-long investigation with this device in four epileptic dogs. Each dog experienced a variety of seizure types after reaching a stable state, with significant temporal variance between them. Cluster seizures were more common than solitary seizures, which were rare (68).

Up to 14 months of continuous EEG recording in epileptic dogs comprised the huge archive of continuous data studied for this project, necessitating the use of rigorous, automated approaches, including machine learning, for the detection and analysis of EEG activity. The results confirmed canine epilepsy as a promising human epilepsy model and produced an unprecedented set of continuous iEEG data for research, including crowdsourcing competitions (27, 81). Patients who suffer from epileptic seizures may live better lives if epileptic seizures can be predicted using machine learning algorithms working with iEEG or scalp EEG data (82, 83), which may apply to both people and DWE as well as caregivers of DWE.

The absence of open access to long-duration EEG recordings with sufficient numbers of seizures to allow researchers to objectively compare algorithms and findings, however, has slowed down the development of effective seizure forecasting. Using a combination of short-term human iEEG data (942 seizures recorded over >500 days) and long-term canine iEEG data (348 seizures recorded over 1,500 days), a significant international seizure prediction competition was conducted in 2014 (27). The results of these investigations showed that seizure forecasting in canine and human epilepsy was feasible. Since then, deep learning algorithms created for seizure forecasting have been improved using long-duration iEEG recordings from epileptic dogs collected by the NeuroVista SAS or other iEEG devices (79, 84–86). Long-term recordings of epileptic dogs provide some of the strongest evidence that seizure prediction is possible, supporting the validity of canine epilepsy as a translational model (3). These studies have demonstrated that seizure prediction outperforms chance in all tested dogs.

### CorTec's Brain Interchange device (https://www.cortec-neuro.com/products-and-services/brain-interchange-one/)

CorTec (Freiburg, Germany) designed an adaptive neuromodulation system, the Brain Interchange (BIC), for continuous electrophysiology recording and programmable electrical stimulation in PWE. The CorTec BIC device includes a fully implantable, hermetically sealed electronics acquisition device for 32-channel stimulation and recording (1 kHz bandwidth; 16 bit, 74 nV resolution). Electrical stimulation of the 32 electrodes is performed with programmable stimulation paradigms. The implanted device amplifies, filters, and digitizes the recorded EEG. The implant is inductively powered by an external unit that communicates with the implant *via* a broad-band radio link. The system is designed within FDA requirements (e.g., thermal monitoring, limitation of stimulation current/voltage, charge balancing) (87) for recording from the surface and in the depth of the brain. The CorTec BIC was also recently implanted in a canine demonstrating the feasibility of continuous recording and electrical brain stimulation synchronized with behavior, which will be published soon.

## Non-invasive EEG systems

Mobile or ambulatory scalp EEG systems can be used in people for continuous EEG monitoring over several days (88, 89), but—as discussed above—scalp EEG-based systems are not usable in dogs. In epileptic dogs, subcutaneous needle electrodes placed under sedation or anesthesia were used for recording wireless ambulatory EEG with synchronized video (75), but the duration of EEG recording was restricted to a few hours.

A novel, wireless, wearable single-channel EEG sensor (Epilog™) has been developed by Epitel (Salt Lake City, UT, USA) for PWE. When applied below the hairline, the Epilog miniature EEG sensor uses single-use disposable “stickers” that act as both the adhesive and the conductive hydrogel that forms the interface between Epilog and the scalp in people (90). The epileptologist's guidance is used to determine the best sensor placement for several scalp locations based on data from an initial seizure diagnosis, including seizure semiology, imaging, and EEG. Data is extracted from the sensor's onboard memory and data are read *via* the Persyst^®^ software (Solana Beach, CA, USA), a common EEG reviewing platform. In a clinical study in which epileptologists blindly reviewed single-channel EEG, from both wired-EEG and Epilog sensors, seizures were accurately identified in 71% of Epilog recordings and 84% of single-channel wired recordings (90). Thus, single-channel EEG performed better than patient self-reporting in diaries based on the literature. Although the Epilog device is of potential interest for EEG recording in dogs, the limitations of scalp EEG recordings in dogs discussed above restrict its utility.

## Conclusions

**Figure 6:**
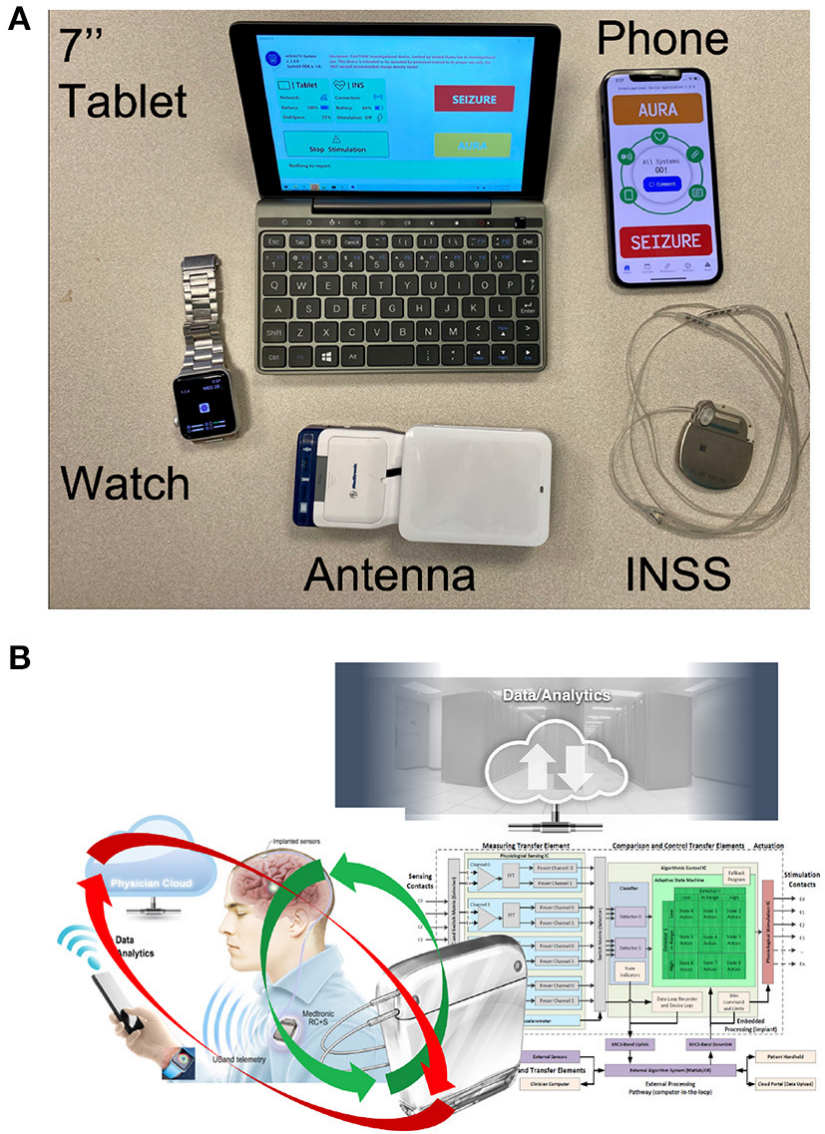
The components of the next generation epilepsy system. **(A)** Tablet computer and smartphone running epilepsy patient assistant (EPA). The EPA application integrates communication between all hardware elements (smartwatch, tablet, phone, and implanted neural sensing and stimulation (INSS) device). The system requires a proprietary antenna to communicate between the INSS and the tablet. **(B)** The brain co-processor system schematic shows the interaction between the implanted device, smartphone, smartwatch, and cloud computing environment (adapted from Kremen et al. (91)).

With ubiquitous digital technology, EEG is no longer just a routine 20-min recording without video but, as described here, several devices are available that allow continuous (24/7) EEG monitoring over long periods. Based on the rapid advances in technology, next-generation epilepsy systems will be available soon (Figure 6), including artificial intelligence or machine learning algorithms for improved seizure detection. For the epilepsy specialist, prolonged video-EEG monitoring is the gold standard, because the combination of prolonged EEG and video leads to an increase in the yield of captured paroxysmal events and interictal discharges (12). This allows answering whether the paroxysmal events are epileptic, to which seizure semiology and type of epilepsy they belong, and, if focal, where the likely focus is located. All approved and investigational EEG devices discussed here have been developed for humans. However, as demonstrated by the numerous canine studies on iEEG devices, these devices can be used in dogs. This also applies for at least some of the novel subscalp EEG devices such as exemplified for the 24/7 EEG™ SubQ system from UNEEG Medical. We hope to present the first canine data with this system soon. We anticipate that the use of such systems in dogs will revolutionize the diagnosis of canine epilepsy and significantly contribute to an improved classification scheme. We also foresee that prolonged EEG monitoring in dogs, both in the inpatient and ambulatory setting, will guide and improve the treatment of epilepsy in this species. Although the costs of the novel EEG devices are a limiting factor for routine use in veterinary medicine, such devices may be particularly useful for dogs with infrequent seizures and the therapeutic management of dogs with drug-resistant epilepsy.

However, there remains the problem of persuading animal keepers to use implantable EEG devices in their pets, which may be a possible limitation of the use of implant technology in veterinary medicine in every-day patients. In this respect, the argument that EEG can help with classification and drug selection is important to consider when evaluating the balance of risk, and the potential for reducing seizures vs. the risk of injury and death with recurrent seizures. An undoubted advantage would be the development of a standardized EEG or iEEG acquisition protocol and demonstrating the benefits of such diagnostics by proving the unequivocal use of the results of EEG analysis in the development of a targeted (customized) treatment of epileptic seizures in dogs. Concerning ethical aspects of using implanted devices in DWE, these aspects may be framed within the unequivocal evidence that EEG improves the therapeutic management of humans with epilepsy. While this is not proven in canines it is likely the case that to optimize drug therapy accurate seizure counts and classification are needed, just like in human epilepsy. We argue that given the potential benefit from the acquired information and the low risk can justify the application of EEG to canines.

We believe there is an opportunity for collaboration between physicians caring for humans and veterinarians caring for pet dogs to help translate the rapid developments in neurotechnology into new and improved treatments for patients.

## Author contributions

WL planned manuscript scope and wrote the first draft. GW reviewed, extended, and edited the draft. All authors approved the submitted version.

## Funding

WL's own studies in dogs (LO 274) have been funded in part by the Deutsche Forschungsmeinschaft (Bonn, Germany). GW research in canines with epilepsy was supported by National Institutes of Health (NIH) R01- NS092882 (*Reliable Seizure Prediction using Physiologic Signals and Machine Learning*) and UH2/UH3 NS95495 (*Neurophysiologically Based Brain State Tracking and Modulation in Focal Epilepsy*). This Open Access publication was funded by the Deutsche Forschungsgemeinschaft within the program LE 824/10-1 Open Access Publication Costs and University of Veterinary Medicine Hannover, Foundation.

## Conflict of interest

Author WL declares that the research was conducted in the absence of any commercial or financial relationships that could be construed as a potential conflict of interest. Author GW declares intellectual property licensed to NeuroOne Inc. & Cadence Neuroscience.

## Publisher's note

All claims expressed in this article are solely those of the authors and do not necessarily represent those of their affiliated organizations, or those of the publisher, the editors and the reviewers. Any product that may be evaluated in this article, or claim that may be made by its manufacturer, is not guaranteed or endorsed by the publisher.
